# Comparisons of therapeutic outcomes in patients with nonampullary duodenal neuroendocrine tumors (NADNETs): A multicenter retrospective study

**DOI:** 10.1097/MD.0000000000016154

**Published:** 2019-06-28

**Authors:** Seung Woo Lee, Jae Kyu Sung, Young Sin Cho, Ki Bae Bang, Sun Hyung Kang, Ki Bae Kim, Sae Hee Kim, Hee Seok Moon, Kyung Ho Song, Sun Moon Kim, Il-Kwun Chung, Dong Soo Lee, Hyun Yong Jeong, Sei Jin Youn

**Affiliations:** a Division of Gastroenterology, Department of Internal Medicine, College of Medicine, Daejeon St. Mary's Hospital, The Catholic University of Korea; b Department of Internal Medicine, Chungnam National University, College of Medicine; c Division of Gastroenterology, Department of Internal Medicine, Soonchunhyang University College of Medicine, Cheonan Hospital, Cheonan; d Department of Internal Medicine, Dankook University College of Medicine; e Department of Internal Medicine, Chungbuk National University School of Medicine; f Department of Internal Medicine, College of Medicine, Eulji University; g Department of Internal Medicine, College of Medicine, Konyang University, Republic of Korea.

**Keywords:** duodenum, endoscopic resection, neuroendocrine tumor, surgery, treatment

## Abstract

Duodenal neuroendocrine tumors (DNETs) are rare tumors that are occasionally found during upper endoscopies. The incidence of DNETs is increasing, although the data regarding treatment outcomes are insufficient. The aim of this study was to evaluate the treatment outcomes in patients with nonampullary DNETs who underwent endoscopic resection or surgery. We evaluated the medical records of patients who were diagnosed with nonampullary DNETs from 2004 to 2017 in 7 university hospitals. We retrospectively analyzed clinical characteristics and compared therapeutic outcomes based on the endoscopic lesion size and treatment method. We ultimately enrolled 60 patients with nonampullary DNETs who underwent endoscopic and surgical treatments. In the endoscopic treatment group, the en bloc resection, endoscopic complete resection (CR) and pathologic CR rates were 88%, 92%, and 50%, respectively. The endoscopic treatment group was divided into 3 subgroups based on the lesion size (1–5 mm, 6–10 mm, and ≥11 mm). The pathologic CR rate was significantly lower in the subgroup with a lesion size ≥11 mm (0%, *P* = .003) than those in the other 2 subgroups. Lymphovascular invasion occurred significantly more frequently (33.3%, *P* = .043) among those with a lesion size ≥11 mm. The pathologic CR rate in the surgical treatment group was higher (90.9%) than that in the endoscopic treatment group (50%, *P* = .017). Surgical treatment appears to be a more appropriate choice because of the risks of incomplete resection and lymphovascular invasion after endoscopic treatment for lesions larger than 11 mm.

## 1 Introduction

Duodenal neuroendocrine tumors (DNETs) are rare neoplasms that are occasionally found during upper gastrointestinal endoscopies. The overall incidence of DNETs is 0.19/100,000 in the United States^[1]^ and these tumors account for 2.0% of all digestive NETs.^[2]^ Importantly, the incidence of these tumors has shown an increasing trend.^[3]^ DNETs can be divided into ampullary and nonampullary DNETs based on their location. The most common treatment for periampullary DNETs is surgery, but nonampullary DNETs can be treated endoscopically or surgically depending on their size.^[4]^ Currently, treatment guidelines for 1 to 2 cm tumors have not been defined, which is partly due to insufficient data regarding the treatment outcomes of nonampullary DNETs. Additionally, 2 large-scale studies have reported conflicting assessments of the safety of endoscopic resection.^[5,6]^ Therefore, it is necessary to analyze the overall treatment outcomes of DNETs based on the endoscopic lesion size and treatment options. The aim of this study was to analyze and compare the treatment outcomes of patients with nonampullary DNETs who underwent endoscopic or surgical resection.

## 2 Materials and methods

### 2.1 Patients

**Figure 1:**
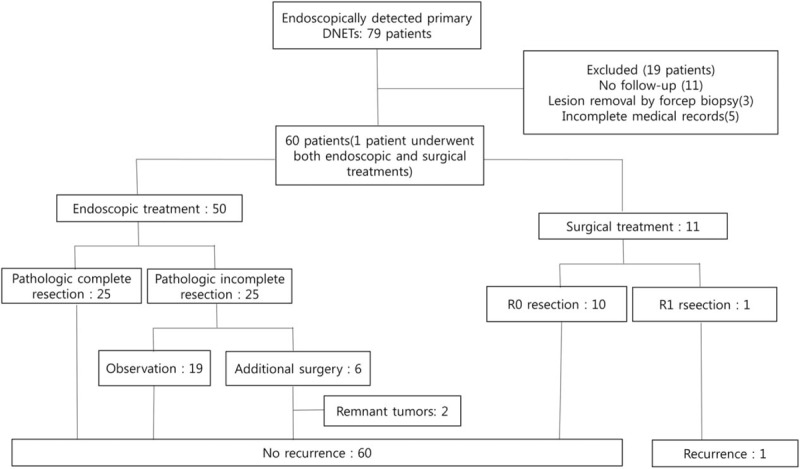
Flow chart of DNETs treated with endoscopy and surgery.

This was a retrospective study conducted in 7 university hospitals in Daejeon-Chungcheong Province in South Korea. The medical records of patients diagnosed with nonampullary DNETs from 2004 to 2017 were analyzed. All of the patients with nonampullary DNETs who underwent endoscopic or surgical resection during this period were included. Cases with no endoscopic follow up, complete removal after forceps biopsy and incomplete medical records were excluded. Seventy-nine patients were identified, and 19 were excluded (Fig. 1). One patient underwent surgical resection and endoscopic resection consecutively. We analyzed this patient based on the 2 separate treatments and therefore allocated the patient to both the endoscopic and surgical treatment groups. We ultimately analyzed 50 endoscopically treated and 11 surgically treated patients. The study was approved by the institutional review board of each of the participating centers in accordance with the *Declaration of Helsinki*.

### 2.2 Definitions

En bloc resection and endoscopic complete resection (CR) indicate total resection of the tumor in 1 piece and no visible remnant tumor at the resection site, respectively. Pathologic CR indicates no lateral and vertical resection margin involvement and no lymphovascular invasion. R0 resection is equivalent to pathologic CR, and R1 resection indicates the possibility of a microscopic tumor remnant in the surgical specimens. Endoscopic morphology was defined based on the Paris classification.^[7]^ The procedure time in the endoscopic treatment group was defined as the time from circumferential marking to the end of hemorrhage control. The procedure time in the surgical treatment group was defined as the duration of general anesthesia.

The histological grades were defined as grades 1, 2, or 3 based on the mitotic index and the Ki-67 index, as defined in the 2010 WHO classification.^[4]^ We reviewed previous pathology slides generated before 2010 that did not have a reported histological grade.

### 2.3 Endoscopic and surgical treatments

**Figure 2:**
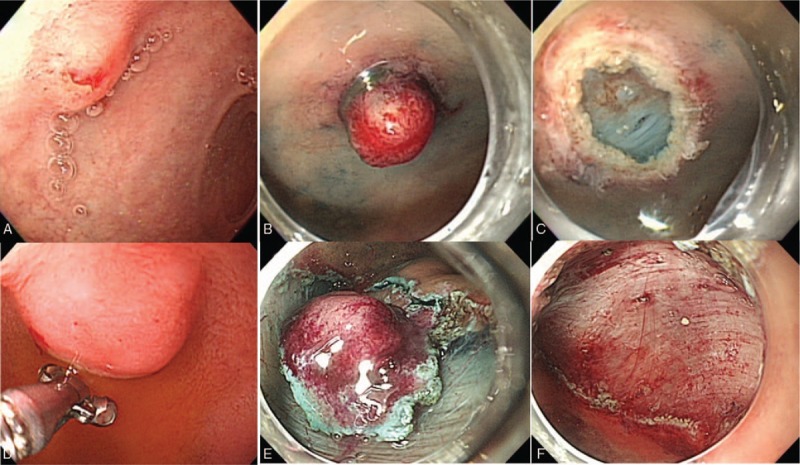
Endoscopic images. A. A 6-mm DNET with dimpling on the surface was detected on the anterior wall of the duodenal bulb. B. The lesion was captured by a rubber band. C. Endoscopic resection was performed by snaring. No remnant tissue was observed endoscopically. D. A 15-mm DNET was detected on the lesser curvature of the duodenal bulb. E. Circumferential mucosal incision was performed. F. ESD was completed. No remnant tissue was observed endoscopically.

Overall, 50 endoscopic treatments were performed by experienced endoscopists in each hospital. Several endoscopic treatment methods were used: Endoscopic mucosal resection with a dual channel endoscope (EMR-D), EMR after band ligation (EMR-L), EMR with a transparent cap (EMR-C), EMR with circumferential mucosal precutting (EMR-P) and endoscopic submucosal dissection (ESD). The procedures were conducted according to the methods described by the American Society for Gastrointestinal Endoscopy (ASGE) committee.^[8]^ EMR-D was performed using an alligator forceps and a snare with a 2-channel endoscope (GIF-2T240or GIF-2TQ260M, Olympus Optical, Japan). The other EMR and ESD procedures were performed with a single-channel endoscope (GIF-H260, GIF-H290, HQ290, Olympus, Japan). All of the procedures were performed with the patient under conscious sedation or sedation by an anesthesiologist in an endoscopy room. The knife used during EMR-P was a Dual Knife (KD650Q, Dual Knife, Olympus, Japan) or an IT knife nano (KD-612L, Olympus, Japan). ESD procedures were performed by marking the incisional area, making a circumferential mucosal incision and then dissecting the submucosa using a Dual knife or an IT knife nano. Examples of endoscopic resections are shown in Figure 2.

Eleven surgical treatments were performed, including 8 wedge excisions, 2 segmental resections and 1 subtotal gastrectomy. All of the surgical procedures were performed with the patient under general anesthesia in an operating room.

### 2.4 Statistical analysis

The data were analyzed using SPSS version 18.0 (SPSS Inc., Chicago IL). Continuous data were evaluated using the Mann–Whitney *U* test or the Kruskal–Wallis test. Categorical variables were examined using the *χ*^2^ or Fisher exact test. *P* values less than .05 were considered statistically significant.

## 3 Results

### 3.1 Demographic data (Table 1)

The mean patient ages were 61.6 years and 58.3 years for endoscopically and surgically treated patients, respectively, and more male patients (52% and 81.8%, respectively) than female patients were present in both groups. The patients usually had no symptoms (80% and 91%, respectively), and those who were symptomatic had no carcinoid-related symptoms. The reported symptoms were epigastric pain, epigastric soreness, dyspepsia and abdominal discomfort. The underlying diseases were hypertension, diabetes mellitus, cardiovascular disease, cerebrovascular disease, and cancer. Most of the lesions were located in the duodenal bulb (90% and 81.8%, respectively) and were singular (100% and 90.9%, respectively) in both groups. The most common morphology was the Is type (66% and 72.7%, respectively) as determined by the Paris classification in both groups.^[7]^ More overlying mucosal abnormalities were noted in the surgical treatment group (54.5%) than in the endoscopic treatment group (32%).

**Table 1:**
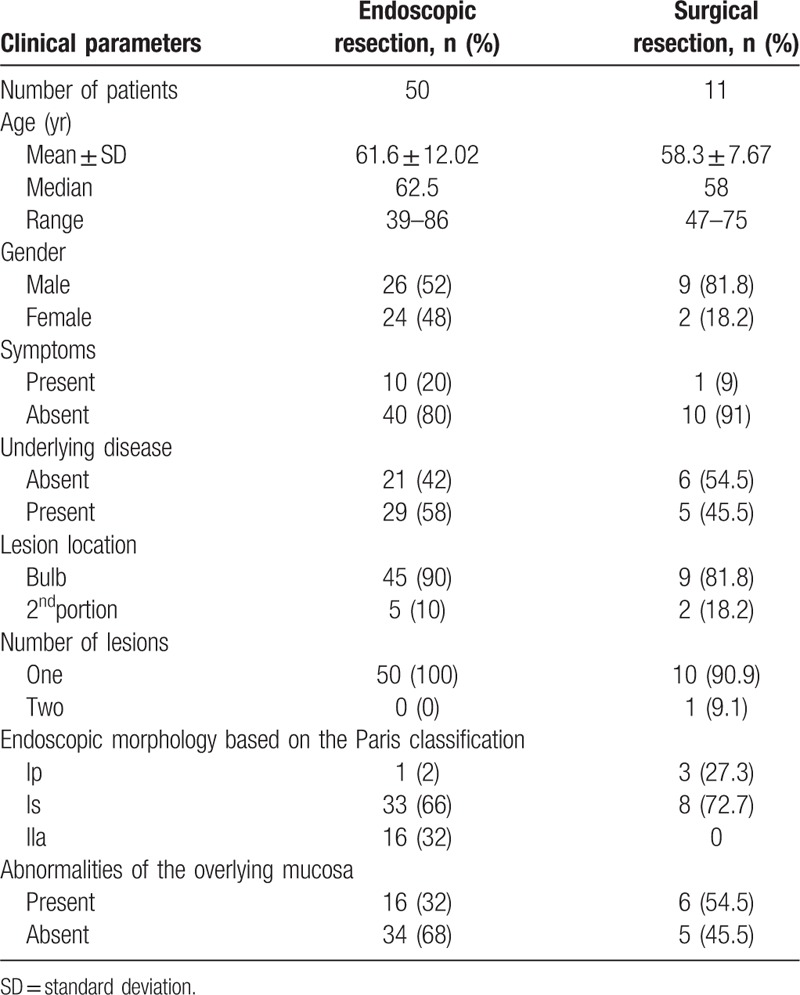
General information.

### 3.2 Therapeutic outcomes according to endoscopic lesion size in the endoscopic treatment group (Table 2)

We divided the endoscopic treatment group into 3 groups according to lesion size: 1 to 5 mm (group 1), 6 to 10 mm (group 2), and ≥11 mm (group 3). No differences were found among the 3 groups in terms of the rates of en bloc resection or endoscopic CR. The pathologic CR rate was significantly lower in group 3 (0%, *P* = .003) than those in groups 1 and 2. The tumor grades were not significantly different among the 3 groups; however, the percentage of grade 2 tumors gradually increased from group1 (7.1%) to group 3 (22.2%). Lymphovascular invasion occurred significantly more frequently in group 3 (33.3%, *P* = .043).

**Table 2:**
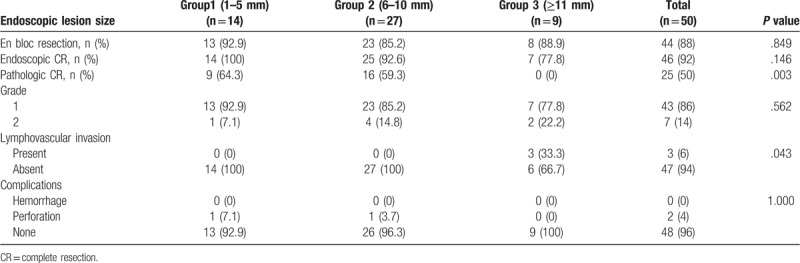
Comparison of therapeutic outcomes according to DNET size.

### 3.3 Procedure-related parameters in the endoscopic treatment group (Tables 3 and 4)

Five endoscopic methods were used for the treatment of nonampullary DNETs. The procedure time was longest in the ESD group (*P* = .002), and the endoscopic lesion size (*P* = .046) and resected specimen size (*P* = .009) were larger in the ESD group than those in the other procedure groups. The rates of en bloc and endoscopic CR were not significantly different among the procedure groups. The pathologic CR rate was higher in the EMR-C (83.3%) and the EMR-P (80%) groups than in the other procedure groups (*P* = .040). No patients in the ESD group achieved pathologic CR (0%). No hemorrhagic complications were identified, although 2 perforations (4%) were noted, which were closed by clipping and treated. The detailed information of the surgically treated patients is shown in Table 4.

**Table 3:**
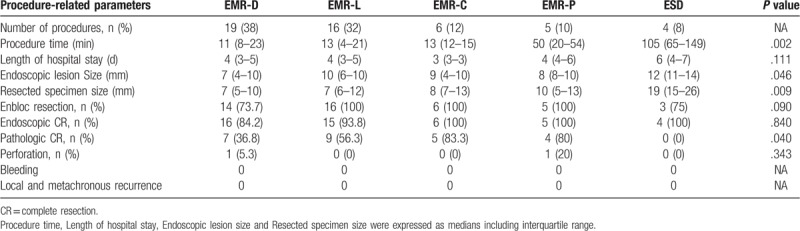
Comparison of procedure-related parameters among different endoscopic procedures.

**Table 4:**
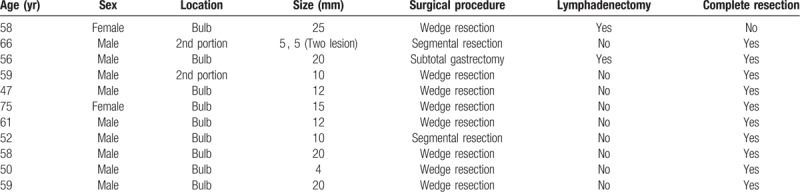
Information of surgically treated patients.

### 3.4 Comparison between endoscopic resection and surgical resection (Table 5)

The procedure time (*P* = .000) and length of hospital stay (*P* = .000) were longer in the surgical treatment group than in the endoscopic treatment group. In addition, the endoscopic lesion size (*P* = .005) and resected specimen size (*P* = .000) were larger in the surgical treatment group.

**Table 5:**
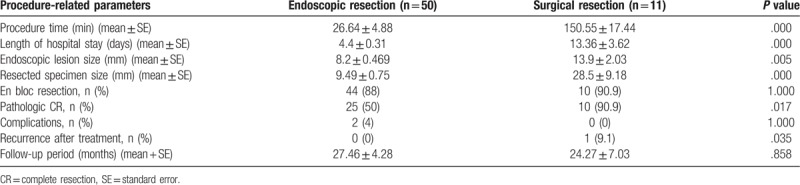
Comparison of endoscopic and surgical resection.

The pathologic CR rate was higher in the surgical treatment group (90.9%) than that in the endoscopic treatment group (50%) (*P* = .017), although the mean endoscopic lesion size was larger in the surgical treatment group (*P* = .005). In the surgical treatment group, only 1 patient experienced recurrence, and this patient was diagnosed with a grade 3 neuroendocrine carcinoma. The mean follow-up periods and complication rates were similar between the groups.

### 3.5 Cases of pathologically incomplete resection in the endoscopic treatment group (Table 6)

Twenty-five cases of pathologically incomplete resection were among the 50 endoscopic resection cases. The most common reason for incomplete resection was vertical resection margin involvement. Six of the 25 patients underwent additional surgical resection. The additional surgeries performed after incomplete endoscopic resection consisted of 4 wedge resections and 2 subtotal gastrectomies. Two of these 6 patients had remnant tumor tissue after additional surgery; these patients did not achieve endoscopic CR after EMR. The mean follow-up period was 20 months for R1 patients who did not receive additional surgery or endoscopic treatment. These patients received regular check-up examinations and no recurrence was observed. The endoscopic CR rate was lower in the additional surgery group (50%) than that in the observation group (94.7%).

**Table 6:**
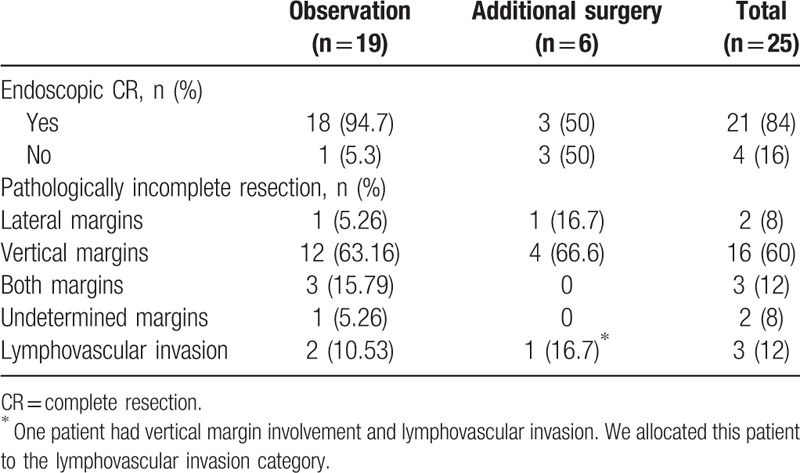
Summary of cases of pathologically incomplete resection in the endoscopic treatment group.

### 3.6 Patients with Grade 2 and Grade 3 NETs

The patient with recurrence was a 58-year-old female, and her initial biopsy results revealed a carcinoid tumor with a lesion size of 25 mm. This patient underwent wedge resection, but lateral and vertical margin involvement, lymphovascular invasion, and lymph node involvement were observed. The final pathologic diagnosis was a grade 3 neuroendocrine tumor (neuroendocrine carcinoma), indicating a pathologic discrepancy between the initial biopsy and final pathology. This patient received chemotherapy but presented with a recurrent liver mass 27 months after surgery. The lesion at the metastatic site was solitary and resectable. The patient underwent local hepatic resection and lived for 61 months. The patients with grade 2 NETs are presented separately in Table 7.

**Table 7:**
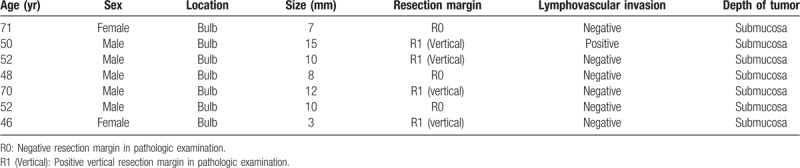
Information of NET Grade 2 patients.

## 4 Discussion

In the present study, we found that NETs ≥11 mm in size are associated with a lower pathologic CR rate and a higher rate of lymphovascular invasion than NETs ≤10 mm in size. Therefore, based on the results of the present study, surgical treatment is suggested in patients with NETs ≥11 mm in size.

The ENETS (European Neuroendocrine Tumor Society) consensus guidelines suggest that treatment of nonampullary DNETs should be divided into 3 categories according to the lesion size.^[4]^ For lesions 10 mm or less in size and those larger than 2 cm in size, endoscopic and surgical treatments are usually considered the optimal treatment modalities, respectively.^[4,5]^ However, the treatment strategy for 1 to 2 cm lesions is controversial and undefined.

The key point when considering endoscopic resection is whether the lesion is completely resectable. The aim of endoscopic treatment is complete (R0) resection. Because NETs have submucosal invasion, complete endoscopic resection can be challenging. The present study showed a low rate (50%) of pathologic CR after endoscopic resection. The difficulty in achieving pathologic CR has been reported in previous studies. One multicenter retrospective study consisting of 38 patients with 41 duodenal NETs ≤ 10 mm reported a 41% pathologic CR rate.^[8]^ Another study including 32 NETs < 20 mm is size reported a 50% pathologic CR rate.^[6]^ In the present study, the pathologic CR rate was significantly lower for ≥11 mm lesions than that for 1 to 5 mm or 6 to 10 mm lesions. An aforementioned study indicated that 10 to 20 mmNETs tend to be associated with decreased pathologic CR rates compared to <10-mm NETs after endoscopic resection.^[9]^ Our results suggest that the complete endoscopic resection rates of ≥11-mm-sized NETs is limited, although drawing a firm conclusion in this regard is difficult given the small number of studies reporting the outcomes of endoscopic resection for DNETs by lesion size.

Prediction of the metastasis risk is an important factor when selecting the treatment method. Tumor size, the depth of invasion, World Health Organization (WHO) grade and lymphovascular invasion are the known risk factors for metastasis. Vanoli et al reported that in grade 2 or 3 proliferative lesions, lymphovascular invasion and invasion beyond the submucosa were significantly associated with local lymph node metastasis.^[9]^ Hatta et al reported that the presence of lymphovascular invasion, multiple tumors, a tumor size of 11 to 20 mm and WHO grade 2 were the risk factors for metastasis.^[10]^ In addition, Untch et al reported that a tumor size ≥1 cm and a high tumor grade were associated with recurrence.^[11]^ In the present study, three lymphovascular invasion cases (6%) were identified among the 50 lesions following endoscopic resection, and all three cases involved tumors ≥11 mm in size (33.3%). Therefore, this study demonstrated that lesions greater than 1 cm in size carry a risk of lymphovascular invasion, which is a risk factor for lymph node metastasis. Furthermore, the proportion of grade 2 classifications tended to increase with increasing tumor size in this study.

The depth of invasion is also an important risk factor for metastasis. Several studies have shown that penetration of the muscularis propria increases the risk for metastasis in nonampullary DNETs.^[12–14]^ Some authors have suggested that if invasion of the muscularis propria on Endoscopic ultrasonogram (EUS) can be ruled out, then the upper size limit for a lesion would only be restricted by the feasibility of endoscopic resection.^[15]^ However, the need for EUS examination in all cases of DNETs, especially those ≤20 mm in size, has not been determined. Indeed, data regarding the depth of invasion based on lesion size in nonampullary DNETs are limited. Therefore, the results of the present study may be useful in establishing the proper therapeutic strategy.

This study compared 5 patient groups according to endoscopic procedure type, including EMR-D, EMR-L, EMR-C, EMR-P, and ESD. Although no differences in the endoscopic CR rates were observed between the groups, the pathologic CR rates were higher in the EMR-P and EMR-C groups than those in the other groups. The EMR-C, EMR-P and ESD groups achieved 100% endoscopic CR rates, but the EMR-D groups achieved only an 84.2% rate. We believe that the cause of this low CR rate in patients who underwent EMR-D may be poor maneuverability in the duodenum. Although ESD for duodenal NET has also been reported to improve the R0 resection rate,^[5]^ the existing data are insufficient. Pathologic CR was not achieved in all cases of ESD in our study. As ESD was selected for lesions >10 mm in size, significant differences in lesion size between the EMR and ESD groups may have affected the CR results. ESD for lesions >1 cm can be inferred to achieve only limited rates of pathologic CR, although the number of such cases in the present study was small. Duodenal ESD is technically difficult and can increase the incidence of surgical complications, such as intraoperative or delayed perforation and hemorrhage.^[16]^ Additionally, the procedure time for ESD is much longer than that for EMR. Furthermore, EMR, including modified techniques (EMR-L, EMR-C, and EMR-P), is faster, technically easier and safer than ESD.^[17]^ No mortality and a low rate of morbidity (4%) were observed in our endoscopic treatment group in contrast to a previous study.^[6]^ Therefore, EMR, including modified techniques, is suggested as the preferred method for resecting DNETs ≤10 mm in size. However, ESD can be used for >10 mm lesions that are difficult to resect en bloc by EMR. In these cases, ESD enables resection of larger tumors compared to EMR and has an advantage of achieving endoscopic CR. The potential requirement for an additional surgery and the risk of incomplete resection should be explained to the patient, and informed consent should be obtained.

No consensus exists regarding treatment decisions for incompletely resected lesions, although the guidelines suggest that surgical resection should be performed when the resection margins are positive.^[4]^ Obtaining pathologic CR for subepithelial tumors is inherently difficult because only a thin layer of normal tissue is present, which can be destroyed during resection. Vertical resection margin involvement was the major cause of pathologically incomplete resection in the endoscopic treatment group in this study. Repeated endoscopic treatment can be considered; however, this strategy is not a simple task due to the presence of fibrosis. As an alternative approach, surgery can be optional though the surgical risks should be considered. Furthermore, the type of surgery that is effective in this case remains unclear. Surgical treatment of all R1 patients may be unnecessary because it is usually possible to discriminate visually whether the residual lesion is present. In our study, of the 50 patients who underwent endoscopic treatment, 25 (50%) had pathologically incomplete resection, and 6 of these 25 patients underwent additional surgery. Three of these 6 patients did not achieve endoscopic CR, 2 of whom had remnant tumors on their surgical specimens. Therefore, endoscopic CR is an important point to consider before performing further treatments.

We followed another 19 of 25 R1 patients who did not receive additional surgery or endoscopic treatment for an average of 20 months, and no recurrence was observed. One study of 13 patients with lesions ≤10 mm in size undergoing a close follow-up without endoscopic or surgical treatment showed no lymph node metastases or tumor-related death during the median follow-up period of 37 months, indicating a favorable natural history of small DNETs.^[18]^ Fitzgerald et al stated that the survival prognosis among patients with DNETs was favorable and reported a 97.9% 5-year disease-specific survival rate for patients with stage I DNETs.^[19]^ Therefore, considering surgical risks, surgery costs and the benign nature of small grade I DNETs, if a lesion ≤10 mm is completely resected endoscopically and the patient has no risk of lymph node metastasis, then management via a close follow-up of deep and lateral resection margin involvement can be an option. Similarly, regular check-up examination was recommended in incomplete resected rectal NETs by endoscopy if lymphovascular invasion were not present in a previous study.^[20]^ However, the final decision whether to perform additional surgery or regular follow-up should be made after consideration of surgical risk and risk factors for metastasis such as lymphovascular invasion, high tumor grade and proper muscle invasion. Further studies are needed to establish a treatment plan in cases of R1 resection.

Our study showed that the pathologic CR rate of surgical treatment was superior to that of endoscopic treatment. If we exclude the patient with a grade 3 NET, the pathologic CR rate was 100%. The complication rates did not differ between the endoscopic treatment and surgical treatment groups. Therefore, we believe that patients with lesions ≥11 mm in size may have better outcomes with surgical treatment. However, the costs and benefits must also be considered when selecting on a treatment plan because the longer procedure time and length of hospital stay were weaknesses of surgical treatment. The most common surgical method was a wedge resection, which showed good results. However, wider resection is needed to be considered in larger lesions (for example, lesions >2 cm) because the possibility of incomplete resection or grade 3 NET exists.

A few limitations exist in this study. First, this study was designed retrospectively. Second, the numbers of grade 2 lesions and 1 to 2 cm lesions were small. Third, the cut-off of 11 mm was not chosen by statistical calculation. However, this cut-off value was selected because there is still controversy whether to perform surgery or endoscopic resection for 11 to 20 mm DNETs.^[4]^ Lastly, DNETs are indolent and slow-growing tumors; therefore, a long-term follow-up is recommended.^[21]^ The follow-up period in this study may have been insufficient to draw reliable conclusions regarding appropriate treatment strategies.

The advantages of the present study are the inclusion of a relatively large number of endoscopically and surgically treated DNETs across multiple centers as well as the evaluation of lymphovascular invasion and tumor grade according to lesion size. These data will be useful for establishing the treatment strategies for DNETs.

In conclusion, modified endoscopic mucosal resection techniques are safe and effective for the treatment of DNETs ≤10 mm in size because of high endoscopic CR rates and the ease of the procedures. For lesions ≥11 mm in size, risks of both incomplete resection and lymphovascular invasion exist, and these lesions tend to be of a higher grade. Therefore, surgical treatment appears to be a more appropriate choice for these lesions than endoscopic treatment. Further studies are necessary to firmly establish proper treatment strategies for all DNETs.

## Author contributions

**Conceptualization:** Seung Woo Lee.

**Data curation:** Seung Woo Lee, Jae Kyu Sung.

**Formal analysis:** Seung Woo Lee.

**Investigation:** Seung Woo Lee.

**Methodology:** Seung Woo Lee.

**Project administration:** Seung Woo Lee.

**Resources:** Seung Woo Lee, Jae Kyu Sung, Young Sin Cho, Ki Bae Bang, Sun Hyung Kang, Ki Bae Kim, Sae Hee Kim, Hee Seok Moon, KyungHo Song, Sun Moon Kim, Il-Kwun Chung, Hyun Yong Jeong, Sei Jin Youn.

**Supervision:** Jae Kyu Sung, Il-Kwun Chung, Dong Soo Lee, Hyun Yong Jeong, Sei Jin Youn.

**Writing – original draft:** Seung Woo Lee, Jae Kyu Sung.

**Writing – review & editing:** Seung Woo Lee, Jae Kyu Sung.
